# Consensus statement on the role of health systems in advancing the long-term well-being of people living with HIV

**DOI:** 10.1038/s41467-021-24673-w

**Published:** 2021-07-16

**Authors:** Jeffrey V. Lazarus, Kelly Safreed-Harmon, Adeeba Kamarulzaman, Jane Anderson, Ricardo Baptista Leite, Georg Behrens, Linda-Gail Bekker, Sanjay Bhagani, Darren Brown, Graham Brown, Susan Buchbinder, Carlos Caceres, Pedro E. Cahn, Patrizia Carrieri, Georgina Caswell, Graham S. Cooke, Antonella d’Arminio Monforte, Nikos Dedes, Julia del Amo, Richard Elliott, Wafaa M. El-Sadr, María José Fuster-Ruiz de Apodaca, Giovanni Guaraldi, Tim Hallett, Richard Harding, Margaret Hellard, Shabbar Jaffar, Meaghan Kall, Marina Klein, Sharon R. Lewin, Ken Mayer, Jose A. Pérez-Molina, Doreen Moraa, Denise Naniche, Denis Nash, Teymur Noori, Anton Pozniak, Reena Rajasuriar, Peter Reiss, Nesrine Rizk, Jürgen Rockstroh, Diana Romero, Caroline Sabin, David Serwadda, Laura Waters

**Affiliations:** 1 Barcelona Institute for Global Health (ISGlobal), Hospital Clínic, University of Barcelona, Barcelona, Spain; 2 University of Malaya, Kuala Lumpur, Malaysia; 3 International AIDS Society (IAS), Geneva, Switzerland; 4 Homerton University Hospital NHS Foundation Trust, London, United Kingdom; 5 Portuguese National Parliament, Lisbon, Portugal; 6 Medizinische Hochschule Hannover (MHH), Hannover, Germany; 7 The Desmond Tutu HIV Centre, Cape Town, South Africa; 8 Royal Free London NHS Trust and University College London, London, United Kingdom; 9 Chelsea and Westminster NHS Foundation Trust, London, United Kingdom; 10 Centre for Social Impact, University of New South Wales, Sydney, Australia; 11 Bridge HIV, San Francisco Department of Public Health, San Francisco, United States; 12 Center for Research in Sexuality, AIDS and Society, Universidad Peruana Cayetano Heredia, Lima, Peru; 13 Fundación Huésped, Buenos Aires, Argentina; 14 Aix Marseille Univ, Inserm, IRD, SESSTIM, Sciences Economiques & Sociales de la Santé & Traitement de l’Information Médicale, ISSPAM, Marseilles, France; 15 Global Network of People Living with HIV (GNP+), Cape Town, South Africa; 16 Imperial College London, London, United Kingdom; 17 University of Milan, Milan, Italy; 18 Positive Voice, Athens, Greece; 19 National Plan on AIDS, Ministry of Health, Madrid, Spain; 20 HIV Legal Network, Toronto, Canada; 21 ICAP at Columbia University, New York City, United States; 22 Spanish AIDS Interdisciplinary Society (SEISIDA), Madrid, Spain; 23 Universidad Nacional de Educación a Distancia, Madrid, Spain; 24 Modena HIV Metabolic Clinic, Università degli studi di Modena e Reggio Emilia, Modena, Italy; 25 King’s College London, London, United Kingdom; 26 Burnet Institute, Melbourne, Australia; 27 Liverpool School of Tropical Medicine, Liverpool, United Kingdom; 28 Public Health England, London, United Kingdom; 29 McGill University Health Centre Research Institute, Montreal, Canada; 30 Department of Infectious Diseases, The University of Melbourne at the Peter Doherty Institute for Infection and Immunity, Melbourne, Australia; 31 Victorian Infectious Diseases Service, The Royal Melbourne Hospital at the Peter Doherty Institute for Infection and Immunity, Melbourne, Australia; 32 Department of Infectious Diseases, The Alfred and Monash University, Melbourne, Australia; 33 Fenway Health and Harvard Medical School, Boston, United States; 34 Infectious Diseases Department, Hospital Universitario Ramón y Cajal, Madrid, Spain; 35 ESA YOUTH 2030, Nairobi, Kenya; 36 City University of New York Graduate School of Public Health and Health Policy, New York City, United States; 37 European Centre for Disease Control and Prevention, Solna, Sweden; 38 London School of Hygiene and Tropical Medicine, London, United Kingdom; 39 Amsterdam University Medical Centers, University of Amsterdam, Amsterdam, Netherlands; 40 American University of Beirut, Beirut, Lebanon; 41 University Hospital Bonn, Bonn, Germany; 42 University College London, London, United Kingdom; 43 Makerere University School of Public Health, Kampala, Uganda; 44 Central and North West London NHS Trust, London, United Kingdom

**Keywords:** Health services, Quality of life, Comorbidities

## Abstract

Health systems have improved their abilities to identify, diagnose, treat and, increasingly, achieve viral suppression among people living with HIV (PLHIV). Despite these advances, a higher burden of multimorbidity and poorer health-related quality of life are reported by many PLHIV in comparison to people without HIV. Stigma and discrimination further exacerbate these poor outcomes. A global multidisciplinary group of HIV experts developed a consensus statement identifying key issues that health systems must address in order to move beyond the HIV field’s longtime emphasis on viral suppression to instead deliver integrated, person-centered healthcare for PLHIV throughout their lives.

## Introduction

Following the introduction of highly effective antiretroviral therapy (ART) in 1996, the global scale-up of ART resulted in substantial declines in AIDS-related morbidity and mortality. By the end of 2019, 67% of the world’s estimated 38 million people living with HIV (PLHIV) had initiated ART, with 59% achieving viral suppression^1^. The life expectancy for PLHIV who are diagnosed early and are able to take continuous ART now approaches that of the general population^2^. Yet, despite viral suppression, PLHIV often report poor well-being and health-related quality of life (HRQoL)^3–7^.

Factors negatively affecting the HRQoL of PLHIV include multimorbidity, drug and alcohol dependence, poverty, social isolation, difficulties disclosing HIV status, and persecution due to discriminatory laws and attitudes^7–14^. HIV-related stigma and discrimination negatively affect the HRQoL of PLHIV through multiple pathways, including social rejection, low self-esteem, and barriers to accessing health and support services^15–17^. These problems call for a broad health system response to the health-related needs of PLHIV. This includes the provision of integrated services for the prevention and management of communicable and non-communicable diseases, along with psychosocial and other support to address common psychological, social, and access challenges. Approaching the healthcare of PLHIV holistically, with decision-making driven by the person’s priorities rather than by a pathogen-specific paradigm, has the potential to yield better overall health outcomes^18^. Comprehensive, multidisciplinary healthcare for PLHIV requires an integrated, person-centered approach. This is likely to enhance HRQoL and contribute to improvements in population-level health across all domains, including infectious and noncommunicable diseases, mental health, and sexual and reproductive health.

Achieving consistent, long-term virological suppression has become a key marker of successful HIV care^19,20^. Whilst access to effective ART for all remains essential, it should be seen as one aspect of a more multifaceted definition of success. The central goal should be integrated, person-centered healthcare that promotes the importance of HRQoL, recognizing the right of all people to enjoy “the highest attainable standard of physical and mental health”^21^. The World Health Organization (WHO) *Global health sector strategy on* *HIV 2016–2021* briefly addresses the non-HIV-specific chronic care needs of PLHIV although without mention of HRQoL^22^. UNAIDS has included a general health target for PLHIV in its strategic guidance for the first time in 2021. It calls for 90% of PLHIV to “have access to integrated or linked services for HIV treatment and cardiovascular diseases, cervical cancer, mental health, diabetes diagnosis and treatment, education on healthy lifestyle counseling, smoking cessation advice and physical exercise”^23^. Recently, national and regional initiatives have promoted a more person-centered HIV care agenda^24,25^. This reflects the global movement towards person-centered care for illness in general. A body of evidence, including studies of PLHIV, exists to underpin the concept and practice, which is largely, but not exclusively, from high-income coutries^26^. Person-centered healthcare must value the social networks of patients, promote quality of life, and reform structurally to improve patients’ experience interacting with the healthcare system, including respect for and protection of human rights. However, there is not yet a common understanding of what the core values and practices of person-centered, holistic care for PLHIV should encompass, or how aspects of this issue may be context-specific.

As a first step in achieving a common understanding, a multidisciplinary expert panel was convened to engage in a Delphi process to develop a consensus statement on the role of health systems in advancing the long-term well-being of PLHIV from a patient-centered perspective. The overarching purpose of the consensus statement is to guide global, regional, national, and subnational stakeholders in improving health system responses to achieve the best possible long-term health outcomes for PLHIV, including HRQoL outcomes. This article reports on the consensus development process and the agreed-upon consensus points.

### Methodology

We employed a standard six-stage Delphi process^27,28^, including the definition of the problem and identification of experts (Stages 1 and 2, concurrently), three survey rounds (Stages 3–5), and actions based on the findings (i.e., endorsement of consensus statements and recommendations; Stage 6)^29^. (Further details of the methodology for this Delphi study are presented in the Supplementary Information.) The research team (J.V.L., K.S.) established an expert panel comprised of 44 individuals with expertise in the long-term health needs of PLHIV. The expert panel encompassed diverse disciplinary and geographical perspectives, as well as wide-ranging lived experiences (Table 1, Table 2). Eleven expert panel members, including two people living openly with HIV, served as members of a steering committee that was tasked with providing conceptual guidance for this project (J.A., R.B.L.*, G.B., G.C., N.D.*, M.J.F., R.H., A.K.*, J.V.L.*, C.S., K.S.) (project co-chairs denoted by *). Three teams of steering committee members led scoping reviews of the literature on multimorbidity (G.B., A.K.), HRQoL (M.J.F., R.H.), and stigma and discrimination (J.A., G.C.) in order to identify priority issues to consider for the consensus statement. Specifically, the main findings from the respective reviews guided decision-making regarding potential consensus points. In particular, issues thought to be difficult to address or inadequately addressed (e.g., measurement of health-related quality of life, approaches to addressing stigma) were deemed important by the steering committee and expert panel members alike to include in the consensus process.

**Table 1:** Expert panel members.

Name	Title/affiliation	Country of origin
Jane Anderson	Consultant Physician, Homerton University Hospital NHS Foundation Trust (United Kingdom)	United Kingdom
Ricardo Baptista Leite	Member of Parliament, Portuguese National Parliament (Portugal)	Portugal
Georg Behrens	Professor of Internal Medicine, Medizinische Hochschule Hannover (MHH) (Germany)	Germany
Linda-Gail Bekker	Director, Professor, The Desmond Tutu HIV Centre (South Africa)	Zimbabwe
Sanjay Bhagani	Consultant Physician, Royal Free London NHS Trust and University College London (United Kingdom)	Kenya
Darren Brown	Physiotherapist, Chelsea and Westminster NHS Foundation Trust (United Kingdom)	United Kingdom
Graham Brown	Associate Professor and Director of Research and Evaluation, Centre for Social Impact, University of New South Wales (Australia)	Australia
Susan Buchbinder	Director, Bridge HIV, San Francisco Department of Public Health (United States)	United States
Carlos Caceres	Professor of Public Health and Director, Center for Research in Sexuality, AIDS and Society, Universidad Peruana Cayetano Heredia (Peru)	Peru
Pedro Cahn	Scientific Director, Fundacion Huesped (Argentina)	Argentina
Patrizia Carrieri	Epidemiologist, Aix Marseille Univ, Inserm, IRD, SESSTIM, Sciences Economiques & Sociales de la Santé & Traitement de l’Information Médicale, ISSPAM (France)	Italy
Georgina Caswell	Head of Programmes, GNP + (South Africa)	Ghana
Graham Cooke	Professor, Imperial College London (United Kingdom)	United Kingdom
Antonella d’Arminio Monforte	Professor of Infectious Diseases, University of Milan (Italy)	Italy
Nikos Dedes	Chair, Positive Voice (Greece)	Greece
Julia del Amo	Director, National Plan on AIDS, Ministry of Health (Spain)	Spain
Richard Elliott	Executive Director, HIV Legal Network (Canada)	Canada
Wafaa M El-Sadr	Professor, ICAP at Columbia University (United States)	Egypt
María José Fuster-Ruiz de Apodaca	Executive Director, Spanish AIDS Interdisciplinary Society (SEISIDA) (Spain)	Spain
Giovanni Guaraldi	Associate Professor, Modena HIV Metabolic Clinic - Università degli studi di Modena e Reggio Emilia (Italy)	Italy
Tim Hallett	Professor, Imperial College London (United Kingdom)	United Kingdom
Richard Harding	Professor, King’s College London (United Kingdom)	United Kingdom
Margaret Hellard	Professor and Deputy Director, Burnet Institute (Australia)	Australia
Shabbar Jaffar	Professor, Liverpool School of Tropical Medicine (United Kingdom)	Pakistan
Meaghan Kall	Principal Epidemiologist, Public Health England (United Kingdom)	United States
Adeeba Kamarulzaman	Professor of Medicine, University of Malaya; International AIDS Society (IAS) President (Malaysia)	Malaysia
Marina Klein	Professor of Medicine, McGill University Health Centre Research Institute (Canada)	Canada
Jeffrey V Lazarus	Professor, Barcelona Institute for Global Health (Spain)	United States
Sharon R Lewin	Director, The Peter Doherty Institute for Infection and Immunity (Australia)	Australia
Ken Mayer	Professor, Fenway Health and Harvard Medical School (United States)	United States
Pepe Pérez Molina	Attending Physician, Infectious Diseases Department, Hospital Universitario Ramón y Cajal (Spain)	Spain
Doreen Moraa	Communication Executive, ESA YOUTH 2030 (Kenya)	Kenya
Denise Naniche	Research Professor, Barcelona Institute for Global Health (Spain)	France
Denis Nash	Distinguished Professor of Epidemiology, City University of New York Graduate School of Public Health and Health Policy (United States)	United States
Teymur Noori	Expert HIV, European Centre for Disease Control and Prevention (Sweden)	Sweden
Anton Pozniak	Consultant Physician, Chelsea and Westminster NHS Foundation Trust and London School of Hygiene and Tropical Medicine (United Kingdom)	United Kingdom
Reena Rajasuriar	Associate Professor, University of Malaya (Malaysia)	Malaysia
Peter Reiss	Professor of Medicine, Amsterdam University Medical Centers, University of Amsterdam (Netherlands)	Ethiopia
Nesrine Rizk	Assistant Professor, American University of Beirut (Lebanon)	Lebanon
Jürgen Rockstroh	Head of Infectious Diseases, University Hospital Bonn (Germany)	Germany
Caroline Sabin	Professor, University College London (United Kingdom)	United Kingdom
Kelly Safreed-Harmon	Researcher, Barcelona Institute for Global Health (Spain)	United States
David Serwadda	Professor, Makerere University School of Public Health (Uganda)	Uganda
Laura Waters	Consultant Physician in Sexual Health and HIV, Central and North West London NHS Trust (United Kingdom)	United Kingdom

**Table 2:** Expert panel demographic composition and level of engagement.

Characteristic	Count
Gender	
Man	22
Woman	22
Primary sector of employment	
Academic	28
Civil society	4
Public	12
Primary field or area of work	
Healthcare provider	8
Clinical research	20
Non-clinical research	8
Advocacy	4
Other: public health	2
Other: health policy	2
Delphi process engagement	
Round 1 survey	38
Round 2 survey	38
Expert Panel meeting	27
Round 3 survey	40
Participation in one or more components	44
Mean # components engaged in	3.0

The research team drew on health system-related issues identified in the scoping reviews to develop an initial set of 29 proposed consensus points with input from steering committee members. Expert panel members were then asked to indicate agreement or disagreement with consensus points in three survey rounds using the Delphi methodology, with further input collected via qualitative comments on each draft point. In addition, their views were sought on selected topics at an online meeting between the second and third survey rounds.

## Results

Overall, agreement consistently increased for the consensus points across survey rounds, which is likely indicative of the incorporation of modifications based on the open-ended comments into the final two rounds. In the third survey round, expert panel members reported unanimous agreement with 22 of the 31 items, and greater than 90% agreement with the remaining nine items (Table 3). In only three of the final 31 points did we observe a somewhat different pattern: point 2.4 had a slight shift from ‘Agree’ to ‘Somewhat agree’; point 4.1 had slight shifts from ‘Agree’ *and* ‘Disagree’ to ‘Somewhat agree’; and point 1.3 had a slight shift from ‘Agree’ to ‘Disagree.’ For these three points, however, the aggregate ratings were still strongly in agreement. (See the Supplementary Information for an explanation of the consensus grading rubric employed.)

**Table 3:** Consensus points on the role of health systems in advancing the long-term well-being of people living with HIV (PLHIV).

Point	Grade	A(%)	SA(%)	SD(%)	D(%)
**1. Framing a more comprehensive health agenda for people living with HIV (PLHIV)**					
1.1. PLHIV at all stages of their lives face unique health challenges. Therefore, health systems need to provide comprehensive healthcare for PLHIV and focus on additional outcomes beyond virological suppression.	U	100			0
1.2. The World Health Organization’s definition of health as “a state of complete physical, mental and social well-being and not merely the absence of disease or infirmity” as well as the Sustainable Development Goal of “[ensuring] healthy lives and [promoting] well-being for all at all ages” underscores the need for healthcare paradigms that look beyond HIV-focused care to address the overall health and well-being of PLHIV in an integrated, people-centered manner.	A	97.5			2.5
1.3. New clinical and public health targets are needed in all countries, regardless of economic status, in order to optimize health system resources, achieve better health engagement, and improve health outcomes in PLHIV.	A	95			5
1.4. Respecting, protecting, and fulfilling human rights is a necessary element of a strong, effective response to HIV, and this approach should be embodied in all efforts to address the long-term health needs of PLHIV. This approach encompasses attention to service delivery, structural inequalities, and laws, policies, and practices that affect the health and well-being of PLHIV.	U	100			0
1.5. Actions to advance the health of PLHIV should be equitable, with consideration of the disproportionate burden of HIV among key populations and of the intersectional nature of the stigma and discrimination experienced by many members of these populations.	U	100			0
1.6. Efforts to improve healthcare for PLHIV should be aligned with efforts to expand universal health coverage in accordance with the 2019 United Nations Political Declaration on Universal Health Coverage and related global commitments.	U	100			0
1.7. In accordance with the Greater Involvement of People Living with HIV (GIPA) Principle and the World Health Organization Framework on Integrated People-Centered Health Services, PLHIV should be meaningfully involved in the planning, implementation, and monitoring of all interventions to safeguard and improve their health.	U	100			0
**2. Multimorbidity**					
2.1. Both biological and non-biological factors put PLHIV at higher risk than people without HIV for a range of health concerns, including infectious and non-communicable diseases, resulting in a greater multimorbidity burden in PLHIV.	U	100			0
2.2. Mental health comorbidities are highly prevalent among PLHIV worldwide, along with physical health comorbidities. They both warrant urgent attention from policy-makers and service providers.	U	100			0
2.3. Integrated approaches to multimorbidity prevention and management can reduce current health system inefficiencies and facilitate greater engagement and retention in care.	A	97.5			2.5
2.4. Addressing multimorbidity as a component of comprehensive long-term care for PLHIV entails considering issues reported by PLHIV to undermine their health-related quality of life (HRQoL), such as pain, fatigue, sleep disturbance, and episodic and chronic disability.	U	87.5	12.5	0	0
2.5. Integrated, evidence-based, people-centered models of care for the prevention and management of multiple conditions in PLHIV should provide multidisciplinary team care and effective referral to psychosocial health services.	A	97.5			2.5
2.6. Global monitoring of national HIV responses with regard to comorbidities should include the collection and reporting of disaggregated data for a range of infectious and non-communicable diseases.	A	97.5			2.5
2.7. In their HIV monitoring activities, national health systems should include not only comorbidities specified in global HIV monitoring, but also other comorbidities that are relevant to their particular national contexts.	U	100			0
2.8. Causes of mortality among PLHIV should be monitored at the national level using uniform coding for comparative purposes.	A	97.5			2.5
2.9. Health system data on patterns of morbidity and causes of mortality in PLHIV should be published and used to guide nationally targeted efforts to improve multimorbidity prevention and care in this population.	U	100			0
**3. Self-reported health-related quality of life (HRQoL)**					
3.1. HRQoL is a central aspect of long-term health and well-being. Improving the HRQoL of PLHIV has the potential to improve various outcomes including medication adherence, retention in care, and ultimately clinical outcomes.	U	100			0
3.2. The effectiveness of healthcare for PLHIV should be assessed using more than biomedical outcomes. Self-reported HRQoL should be recognized as a core outcome in the clinical management of individual patients, as well as in national and global monitoring of health system responses to HIV.	U	100			0
3.3. The use of patient-reported outcome measures (PROMs) in clinical care for PLHIV has a number of potential benefits, including achieving a person-centered approach; using health system resources more efficiently; empowering PLHIV to raise concerns; facilitating discussion of sensitive problems; encouraging greater engagement with services; and promoting treatment adherence.	A	97.5			2.5
3.4. Validated HIV-specific PROMS should be used to measure the HRQoL of PLHIV in clinical practice, with findings informing efforts to address issues that are reported to be undermining HRQoL.	A	97.5			2.5
3.5. Health system monitoring of HRQoL should use both HIV-specific and non-HIV-specific validated PROMs, with the latter enabling comparisons of the HRQoL of PLHIV to that of people without HIV.	U	100			0
3.6. HRQoL outcomes in PLHIV may be influenced by multifactorial causal pathways involving diverse issues such as multimorbidity, frailty, symptom burden, disability, social isolation, financial insecurity, and HIV-related stigma and discrimination, indicating a need for comprehensive, evidence-based, people-centered healthcare models.	U	100			0
3.7. PROMs that capture information about symptoms should be incorporated into HIV clinical care to support patients’ involvement in their own care and to improve recognition of symptoms and problems that may have a negative impact on health and well-being, such as undiagnosed comorbidities or social issues.	U	100			0
**4. Stigma and discrimination**					
4.1. HIV-related stigma and discrimination, including discriminatory laws, as well as the social environment fostered by these laws, constitute major barriers to the successful implementation and uptake of comprehensive care models for PLHIV.	U	85	15	0	0
4.2. Negative consequences of HIV-related stigma and discrimination include reluctance to use health services, medication non-adherence, internalized stigma, depression, emotional distress, social exclusion, and poor HRQoL.	U	100			0
4.3. Promoting the holistic well-being of PLHIV in clinical and community care settings requires efforts to identify specific ways in which stigma and discrimination affect PLHIV and to respond with interventions at the service delivery, health system, and structural levels.	U	100			0
4.4. Effectively countering HIV-related stigma and discrimination requires actions to address its intersectional nature. This means taking into account how HIV status and HIV risk intersect with other factors that may elicit stigma and discrimination, such as age, sexual orientation, gender identity, sex characteristics, race, religion, ethnicity, indigenous status, national origin, migrant status, drug use, disability, incarceration, and sex work.	U	97.5	2.5	0	0
4.5. HIV-related stigma and discrimination are consistently reported in healthcare settings worldwide and present major barriers to service uptake, service delivery, and retention in care, including for non-HIV-specific health needs. This limits the effectiveness of integrated service delivery models that are essential for advancing the overall health and well-being of PLHIV.	U	100			0
4.6. Countering HIV-related stigma and discrimination in healthcare settings requires expanding the evidence base regarding how to effectively address the root causes of these problems and scaling up interventions shown to achieve lasting improvements.	A	97.5			2.5
4.7. All types of healthcare settings should provide patients with information about their rights and should have formal, confidential channels for reporting stigmatizing and discriminatory behaviors and experiences. There should also be mechanisms for redress when violations of human rights have occurred.	U	100			0
4.8. Validated instruments should be utilized to monitor progress toward the achievement of global and national HIV-related stigma and discrimination targets.	U	100			0

Consensus point response categories: A = agree; SA = somewhat agree; SD = somewhat disagree; D = disagree. In the third round (R3) of data collection, three points (2.4, 4.1, and 4.4) retained the 4-point Likert-type scale response categories, while all other points were assessed using the binary response options of agree/disagree.

Grading of consensus responses: U = unanimous (100%) agreement (A + SA); A = 90% to 99% agreement.

Key terms used in the consensus statement are described in Box 1. HIV organizations globally were invited to endorse the final consensus statement (Box 2).

Box 1 Key concepts **Health-related quality of life (HRQoL)**–a construct that reflects an individual’s perceptions of his or her well-being in health-related aspects of life, with the concept of health understood to encompass physical, mental, and social dimensions^123,124^. While there is a lack of consensus regarding how HRQoL should be defined, instruments that measure HRQoL commonly address multiple aspects of physical, mental, and social well-being, such as mobility, pain, anxiety, depression, social functioning, and vitality^124^.

**Health system**–as defined by the World Health Organization, “all organizations, people and actions whose primary intent is to promote, restore or maintain health…. A health system is, therefore, more than the pyramid of publicly owned facilities that deliver personal health services. It includes, for example, a mother caring for a sick child at home; private providers; behavior change programmes; vector-control campaigns; health insurance organizations; occupational health and safety legislation^125^.”

**HIV-related discrimination**–as defined by UNAIDS, “the unfair and unjust treatment (act or omission) of an individual based on his or her real or perceived HIV status. Discrimination in the context of HIV also includes the unfair treatment of other key populations, such as sex workers, people who inject drugs, men who have sex with men, transgender people, people in prisons and other closed settings and, in some social contexts, women, young people, migrants, refugees, and internally displaced people…. Discrimination can be institutionalized through existing laws, policies and practices that negatively focus on people living with HIV and marginalized groups, including criminalized populations^99^.”

**HIV-related stigma**–as defined by UNAIDS, “the negative beliefs, feelings, and attitudes towards people living with HIV, groups associated with people living with HIV (e.g., the families of people living with HIV) and other key populations at higher risk of HIV infection, such as people who inject drugs, sex workers, men who have sex with men and transgender people^99^.”

**Integrated health services**–as defined by the World Health Organization, “health services that are managed and delivered so that people receive a continuum of health promotion, disease prevention, diagnosis, treatment, disease-management, rehabilitation, and palliative care services, coordinated across the different levels and sites of care within and beyond the health sector, and according to their needs throughout their life course^126^.”

**Internalized stigma**–an “individual’s personal acceptance” of the stigma associated with their stigmatized identity^127^.

**Intersectional stigma and discrimination**–a concept “characterize[d by] the convergence of multiple stigmatized identities within a person or group^128^” and discussed by the UNAIDS Global Partnership for Action to Eliminate All Forms of HIV-related Stigma and Discrimination as, “fueled by multiple factors, including their HIV or another health status, age, sex, gender identity, sexual orientation, race, disability, ethnicity, drug use, migration status, etc^80^.”

**Key populations**–as defined by the World Health Organization, “groups who, due to specific higher-risk behaviors, are at increased risk of HIV irrespective of the epidemic type or local context. Also, they often have legal and social issues related to their behaviors that increase their vulnerability to HIV.” The five key populations widely addressed in the global response to HIV are men who have sex with men; people who inject drugs; people in prisons and other closed settings; sex workers; and transgender people^129^.

**Multimorbidity**–the coexistence of multiple (somatic and/or mental) health conditions in addition to HIV.

**People-centered care**–as defined by the World Health Organization, “an approach to care that consciously adopts individuals’, carers’, families’ and communities’ perspectives as participants, and beneficiaries of, trusted health systems that are organized around the comprehensive needs of people rather than individual diseases, and respects social preferences. People-centered care also requires that patients have the education and support they need to make decisions and participate in their own care and that carers are able to attain maximal function within a supportive working environment. People-centered care is broader than patient and person-centered care, encompassing not only clinical encounters but also including attention to the health of people in their communities and their crucial role in shaping health policy and health services^126^.”

**Psychosocial health services**–As defined by the US Institute of Medicine, “psychological and social services and interventions that enable patients, their families, and health care providers to optimize biomedical health care and to manage the psychological/behavioral and social aspects of illness and its consequences so as to promote better health^130^.”

**Vulnerable populations**–As defined by the World Health Organization, “groups of people who are particularly vulnerable to HIV infection in certain situations or contexts, such as adolescents (particularly adolescent girls in sub-Saharan Africa), orphans, street children, people with disabilities and migrant and mobile workers. These populations are not affected by HIV uniformly across all countries and epidemics^129^.”

Box 2 Organizations endorsing the HIV consensus statement to date

**Table:** 

**Organization**	**Location**
Adhara Asociación VIH/SIDA	Spain
AFEW International	Global
AIDES	France
AIDS Action Europe	Europe
AIDSImpact	United Kingdom
Association de Lutte Contre le Sida (ALCS)	Morocco
Australian Federation of AIDS Organisations	Australia
Barcelona Institute for Global Health (ISGlobal)	Spain
The British HIV Association	United Kingdom
Burnet Institute	Australia
Canadian HIV Trials Network, The Canadian Institutes of Health Research	Canada
Canadian International HIV Rehabilitation Research Collaborative (CIHRRC)	Canada
Central Africa International Epidemiology Databases to Evaluate AIDS (CA-IeDEA)	Central Africa
Centre for Social Impact, University of New South Wales	Australia
Children’s HIV Association	United Kingdom and Ireland
City University of New York Graduate School of Public Health and Health Policy	United States
City University of New York Institute for Implementation Science in Population Health (CUNY ISPH)	United States
Country Coordination Mechanism - Tunisia, The Global Fund	Tunisia
The Desmond Tutu Health Foundation	South Africa
Deutsche AIDS Hilfe	Germany
Deutsche Arbeitsgemeinschaft niedergelassener Ärzte in der Versorgung HIV-Infizierter	Germany
East Europe and Central Asia Union of People Living with HIV (ECUO)	Europe and Asia
Elton John AIDS Foundation	Global
European AIDS Clinical Society	Europe
European AIDS Treatment Group (EATG)	Europe
Fenway Health	United States
Frontline AIDS	Global
Fundación Hu*é*sped	Argentina
German AIDS Foundation	Germany
German AIDS Society	Germany
Global Network of People Living with HIV (GNP+)	Global
Global Network of Young People Living with HIV (Y+ Global)	Global
Grupo de Estudio del SIDA-SEIMC (GESIDA)	Spain
Harvard University Center for AIDS Research	United States
The HIV Justice Network	The Netherlands
HIV Legal Network	Canada
HIV Outcomes	Europe
ICAP at Columbia University	United States
Italian Cohort of Antiretroviral Naïve Patients (ICONA) Foundation	Italy
Institute of HIV Research and Innovation	Thailand
The Institute of Social and Preventive Medicine (ISPM), University Bern	Switzerland
The International AIDS Society (IAS)	Global
International Association of Providers of AIDS Care (IAPAC)	Global
International Community of Women Living with HIV (ICW)	Global
International Planned Parenthood Federation (IPPF)	Global
The Italian Conference on AIDS and Antiviral Research	Italy
Kasr Al-Aini HIV and Viral Hepatitis Fighting Group	Egypt
Malaysian AIDS Council	Malaysia
National Agency for Research on AIDS and Viral Hepatitis (ANRS) - Emerging Infectious Diseases	France
National AIDS Trust	United Kingdom
National Association of People with HIV Australia (NAPWHA)	Australia
National Minority AIDS Council (NMAC)	United States
NCD Alliance	Global
The Peter Doherty Institute for Infection and Immunity	Australia
Positive Voice	Greece
Realize	Canada
Rehabilitation in HIV Association (RHIVA)	United Kingdom
Southern Africa International Epidemiology Databases to Evaluate AIDS (SA-IeDEA)	Southern Africa
Spanish AIDS Interdisciplinary Society (SEISIDA)	Spain
Stichting hiv monitoring (SHM)	The Netherlands
STOPAIDS	Global
Terrence Higgins Trust	United Kingdom
TREAT Asia, the Foundation for AIDS Research (amfAR)	Thailand
Treatment Action Group	United States
UNITE Global Parliamentarians Network to End Infectious Diseases	Global
Universidad Peruana Cayetano Heredia	Peru
West Africa International Epidemiology Databases to Evaluate AIDS (WA-IeDEA)	West Africa

### Summary of evidence

#### Multimorbidity

PLHIV worldwide have a greater burden of multimorbidity than people without HIV and this burden increases with age^3,30–32^. Common comorbidities include hepatitis B, hepatitis C, tuberculosis, and aging-associated noncommunicable diseases such as cardiovascular disease, chronic kidney disease, osteoporosis, and cervical, anal, and other cancers^33–37^. Even with access to effective ART, PLHIV are more likely than people without HIV to experience depression and other mental health disorders, including substance use disorders^16^. Irrespective of whether these conditions precede the HIV infection, which may affect prevention and treatment, multimorbidity in PLHIV is associated with higher levels of hospitalization^38^, higher healthcare costs^39^, higher levels of polypharmacy^40^, and lower HRQoL^7,13^.

Factors contributing to the higher multimorbidity burden in PLHIV include late diagnosis of HIV, ART toxicities, and long-term effects of HIV on the immune system^41,42^. Even among PLHIV who achieve long-term viral suppression, chronic immune activation may contribute to the onset of aging-related comorbidities^43,44^. Determinants of health, including social determinants such as poverty, stigma, and discrimination, as well as environmental factors such as criminalization and incarceration put some PLHIV at increased risk of developing comorbidities and having poorer comorbidity outcomes^13,45–47^. Health-related behaviors such as smoking and dependent drug or alcohol use further add to the multimorbidity burden^48^. Difficulty in disclosing a positive HIV status, as well as experiences of stigma and discrimination deeply affect the mental health of PLHIV and introduce barriers to engagement in care, hence increasing the risk of poor health outcomes^15,49^.

Disease-specific approaches to the provision of health services for PLHIV are likely to be inefficient, particularly because it is common for multiple comorbidities to share the same risk factors, resulting in syndemics^50^. Integrated healthcare models tailored to reflect the multimorbidity-related needs of PLHIV in different geographical settings have the potential to deliver better health outcomes, if implemented equitably^51^, and can do so cost-effectively^52^. By addressing the prevention, screening, and management of comorbidities in a person-centered manner, guided by the needs of individual patients, health systems can also respond flexibly to the multidimensional process of aging with HIV, taking into account both chronic and episodic health-related needs^53^. The priorities of PLHIV may differ from those of service providers. For example, PLHIV may be more concerned than providers about issues such as pain and sleep disturbance^54^. Addressing concerns identified by PLHIV will be important for effective treatment and symptom management.

A new paradigm is needed for addressing multimorbidity in PLHIV. The conceptualization of frailty in geriatric healthcare suggests a framework for identifying PLHIV who are at higher risk of poor health outcomes^55^. WHO’s conceptualization of healthy aging as a process that is influenced by both intrinsic capacity and the environment may also provide insights, along with the related concept of functional ability, which emphasizes people’s ability to do what is important to them at different stages of their lives^55,56^. Further research is needed to determine how these approaches can inform the clinical care of PLHIV.

#### Health-related quality of life

Health is defined by WHO as “a state of complete physical, mental and social well-being and not merely the absence of disease or infirmity”^57^, while international law recognizes the human right of all persons to “enjoyment of the highest attainable standard of physical and mental health” and requires States to take measures to progressively realize this right over time^58^. HRQoL is increasingly acknowledged as an important aspect of health. It is commonly assessed by patient-reported outcome measures (PROMs), which reflect people’s subjective perceptions of their health-related experiences^59^. Validated PROMs can be used to systematically quantify people’s HRQoL, allowing for observations about how HRQoL varies among individuals, across groups, and over time. The comprehensive multidimensional nature of PROMs that measure HRQoL makes them a useful tool for determining how well people are responding to the challenges associated with complex chronic health conditions. Incorporating HRQoL monitoring into clinical and community services for PLHIV can provide a person-centered perspective on the effectiveness of interventions, inform health-related decision-making, and identify PLHIV who would benefit from clinical and community services to address modifiable factors that negatively affect HRQoL.

The relevance of HRQoL as a measure of well-being among PLHIV is well-established. PLHIV have reported poorer HRQoL when compared to members of the general population, and also poorer HRQoL than people with other chronic health conditions^3–5^. Poorer HRQoL scores are closely correlated with worse clinical health measures such as low CD4 T-cell count and the occurrence of AIDS-defining events^60–63^. While comorbidities, disability, and pain impair HRQoL^7,64–66^, successful HIV treatment has a positive impact^67,68^. Physical and mental dimensions of HRQoL scores predict viral rebound and all-cause hospitalization^69^. Better scores on the physical dimension of HRQoL predict survival among PLHIV receiving ART, as well as emergency department utilization and hospital discharge rates^70,71^. Enhancing HRQoL has the potential to improve clinical outcomes, adherence, and retention in care^7,60^.

PLHIV report a number of components of HRQoL that are important to them. These include physical (e.g., pain and gastrointestinal issues), cognitive (e.g., memory and sleep), psychological (e.g., anxiety and depression), social (e.g., isolation and intimacy), functional (e.g., independence and ability to perform everyday activities), welfare (e.g., finances and fears regarding immigration status), spiritual well-being (e.g., achieving a sense of peace), and information (e.g., about long-term outcomes about their condition) components^72–74^. However, issues that are important to PLHIV, especially non-physical issues, maybe missed by those providing routine clinical care due to existing service delivery structures and a lack of training about and awareness of these issues^75^. A healthcare approach that focuses solely or primarily on laboratory results may lead PLHIV to feel that the physical and psychosocial concerns that matter to them are not equally important to their healthcare providers^76^. Therefore, validated PROMs used to measure HRQoL should reflect the symptoms and concerns reported by PLHIV and should be culturally appropriate, including for marginalized groups. Several internationally validated instruments, generic and HIV-specific, are currently used to measure HRQoL in PLHIV for general population comparisons, medico-economic assessments, and specific research and clinical purposes (e.g., SF-12v2, MOS-HIV, PROQOL-HIV, WHOQOL-bref)^59^. As both internalized and enacted stigma negatively impact HRQoL^77,78^, some validated instruments, such as PROQOL-HIV^79^, have included a sub-scale measuring stigma as a component of HRQoL.

Potential benefits of using PROMs in routine HIV care have been readily identified by PLHIV and their service providers: achieving a person-centered approach, empowering individuals to raise concerns, feeling that all concerns are heard and taken seriously, facilitating discussion of sensitive problems, increasing engagement with services and treatment adherence, and identifying who is benefiting or not benefiting from care and support strategies^47^.

#### HIV-related stigma and discrimination

PLHIV may experience multiple forms of stigma and discrimination at various levels (e.g., policy and law, health system, and interpersonal) and in different settings (e.g., health sector, education, workplace, the justice system, families and communities, and emergency and humanitarian settings)^80^. Such experiences have direct social and health consequences (e.g., depression, anxiety, substance use, impaired quality of life, social exclusion, internalized stigma)^16^. They also adversely affect decisions regarding prevention behaviors, engagement in care, and uptake of social services^81–84^.

Discriminatory laws that criminalize or otherwise punish key populations, or that prohibit or impede the delivery of specific health services (e.g., affordable medications, harm reduction services, drug dependence treatment, and sexual and reproductive healthcare), or that fail to respect and protect human rights such as privacy or autonomy in medical decisionmaking, constitute major barriers to the successful implementation of models for comprehensive care for PLHIV^85^. Legal and policy frameworks also can harm the material well-being of PLHIV when social security programs do not adequately support those experiencing disability related to HIV or comorbidities, or when HIV or comorbidity services are not reimbursed, resulting in catastrophic health expenditure^86^.

These examples underscore the role of HIV-related stigma as a major driver of health inequities^87^. The stigma associated with real or perceived HIV status, including within healthcare settings, also intersects with other forms of stigma and discrimination related to social identities or characteristics, e.g., race, ethnicity, sexual orientation, gender identity, age, disability, poverty, migrant status, sex work, drug use, and prior or current incarceration^15,88^. This intersectional stigma can adversely affect physical and mental health outcomes^15^. Layered stigma intensifies the intertwined stigma-discrimination-care relationship, which may lead to delayed, sub-optimal, or avoided care^81,82^. The overload caused by suffering intersectional stigmas has adverse effects on both mental health and health behaviors. This means that the health, economic, and social needs of PLHIV suffering structural inequality must be viewed through the prism of intersectionality if there is to be a reduction in the cognitive, social, physical, and moral distancing these groups experience from that of other people^89^.

Examples of stigma and discrimination reported by PLHIV in healthcare settings include excessive infection control precautions taken by providers, longer waiting times, disrespect, negligence, reduced confidentiality, delay or denial of treatment, and poor support services^90^. Two mechanisms act in synergy: reduced care-seeking behaviors for fear of experiencing stigma and discrimination, and the stigmatizing and discriminatory actions of healthcare staff. Secondary stigma, arising from providing care to stigmatized groups, can also affect health providers and modify their attitudes^90^. PLHIV with multimorbidity may be treated in services where HIV-related stigma could be compounded by additional stigma associated with specific comorbidities (e.g., depression or liver cancer^84^) or through lack of experience with PLHIV. Specific integrated or shared care models incorporating specialists in HIV and other staff, and the development of HIV-friendly practices, are promising approaches to providing person-centered, continuous care^91,92^.

Given the pervasiveness of stigma and discrimination directed at PLHIV in healthcare settings, it is crucial to monitor this experience using validated measures. HRQoL instruments including a stigma dimension (e.g., PROQoL)^79^ may provide an option for data collection of routine patient-reported outcomes. Some validated instruments, such as the People Living with HIV Stigma Index 2.0^93^ and the HIV Stigma Scale^94^, which have been validated in various settings around the world with support from PLHIV, monitor longitudinal trends of experienced stigma^59^. These could be complemented with measures of stigma expressed by healthcare providers^95–98^.

Interventions to counter HIV-associated stigma should also be implemented^99,100^. Such interventions have been evaluated^15,101–103^ and can be categorized as: information-based, structural, biomedical, skill-building, empowerment-based, and contact-centered^98^. Interventions may be patient-focused or provider-focused, and it is possible that optimal impact may be achieved when combinations of interventions are employed. However, further evidence on the effectiveness of such interventions is needed. At a minimum, all types of healthcare settings should provide PLHIV with information about their human rights and a channel for reporting behaviors and policies that are discriminatory or otherwise infringe upon rights in this setting.

## Discussion

While continuing to press for the crucial goal of equitable universal access to ART, health systems must also expand the focus of HIV care. This broader approach will enable PLHIV to experience optimal long-term well-being, consistent with the realization of the human right to enjoy the highest attainable standard of physical and mental health. With this goal in mind, the multidisciplinary expert panel developed this consensus statement on the role of health systems in advancing the long-term well-being of PLHIV. The process resulted in 31 items with which expert panel members expressed high levels of agreement. The consensus statement reflects the current state of knowledge about health-related challenges for PLHIV in the key domains of multimorbidity, HRQoL, and stigma and discrimination, as well as addressing psychosocial needs associated with these issues. Proposed measures for improving the long-term health outcomes of PLHIV build on key principles in the fields of HIV and global health, including principles expressed in the Constitution of the World Health Organization^57^, the 2030 Agenda for Sustainable Development^104^, the United Nations Political Declaration on Universal Health Coverage^105^, and the WHO Framework on Integrated, People-Centered Health Services^106^. This consensus statement and the associated recommendations for action have the potential to improve the well-being of PLHIV throughout their lives.

It has long been recognized that respecting, protecting, and fulfilling human rights can yield better health outcomes for PLHIV^49^, yet human rights are contested in ways that undermine many aspects of the HIV response worldwide. As health systems focus more on the long-term care needs of PLHIV, it is imperative to address the human rights challenges that undermine this effort. Discrimination against PLHIV in healthcare settings is one of many such issues that warrant urgent attention^80^. While some matters are determined by actors outside of health system institutions (e.g., lawmakers adopting laws that regulate certain aspects of how health systems operate and how services are delivered), it is within the power and purview of health system actors (institutions and individuals) to influence many structural factors so as to reduce discrimination and other barriers. Therefore, health systems and the people who work within them must recognize and work to eliminate the multiple forms of structural discrimination that undermine the health of PLHIV. They have an important vantage point as observers of the consequences of such barriers for health and HRQoL and an influential position as advocates, with considerable moral and political persuasion. They also bear responsibility for removing such barriers within healthcare settings, for example by promoting initiatives to hire diverse and inclusive workforces, establishing systems to report and investigate discrimination, and providing health services in geographical locations and at times that will facilitate access for marginalized or vulnerable populations. A pervasive and highly problematic form of structural discrimination is HIV criminalization (i.e., the unjust application of criminal law to people living with HIV based solely on their HIV status), alongside the criminalization and stigmatization of various activities associated with HIV risk^107^. Other relevant and pressing human rights issues include privacy and confidentiality, personal autonomy, access to justice, and meaningful participation in health decisionmaking^108^.

Meaningful participation can be advanced by including PLHIV in the development and enactment of policy and programmatic responses, as called for by the widely endorsed Greater Involvement of People Living with HIV Principle^109^. PLHIV have unique insight into the factors that positively and negatively affect their well-being. Their perspectives are essential in the formulation of effective strategies both at the policy and the service delivery levels^110^. The 2021 United Nations Political Declaration on HIV and AIDS called for the expansion of community-led HIV service delivery to comprise at least 30% of testing and treatment-related services by 2025^111^. This target is even more important in the context of improving the long-term health-related outcomes of PLHIV. Community-based health and psychosocial service providers can do more than supplement government and private services; they also are well-suited to identify innovative and cost-effective models of care and to engage marginalized and vulnerable populations^19,110^. Efforts to address the needs of PLHIV in relation to multimorbidity, HRQoL, and stigma and discrimination can be greatly enhanced through the involvement of communities in setting the research and programmatic agendas, as well as in direct service delivery; such activities need to be sustainably supported and financed.

Evidence suggests that living long-term with HIV can bring episodic functional concerns^112^. Continuous assessment in routine care is essential for anticipation, detection, and management of fluctuating concerns. Such care should include HRQoL measures that take a multidimensional approach, reflecting what matters to PLHIV, including concerns such as stigma and social function^72,113^. For self-reported HRQoL outcomes to inform care delivery in a more person-centered manner, health systems and service organizations must pay careful attention to language, culture, and local and individual priorities regarding well-being. Data should be collected and used within a meaningful patient-provider relationship in order to optimize validity and reliability^114^. This has been achieved even in resource-limited settings with poor literacy and advanced HIV infection^115^. Locally developed systems in Africa, for example, have enabled patients to self-report using a “hand-scoring” system^116^. Other systems support community caregivers of people with cancer and HIV in India and African countries to collect self-reported patient outcomes using mHealth, providing data directly to a provider “dashboard” to inform the allocation of staff resources to those most in need^117^. A growing body of evidence indicates that service delivery approaches to HIV care can incorporate attention to patient experiences and concerns in high-prevalence, low-resource areas^85,118^.

The expert panel carried out its work with the understanding that health systems vary substantially across, and often within, countries. Patterns of multimorbidity likewise vary greatly across geographical regions. Monitoring progress in the global HIV response requires a set of standardized indicators of health behaviors and comorbidities that contribute substantially to the global multimorbidity burden^119^. To the extent that resources allow, health systems are strongly encouraged to monitor these behaviors and comorbidities and to solicit input from PLHIV on additional national monitoring priorities^1,120^. Although many national health systems are not currently monitoring HRQoL, stigma, or discrimination among PLHIV, they should, at a minimum, include indicators from the UNAIDS Global AIDS Strategy 2021–2026, in order to standardize monitoring.

In its Global AIDS Strategy, UNAIDS set ambitious 2025 targets. It established 10 result areas that broadly aim to achieve equitable access to fully-resourced and integrated HIV services for all people living with or at risk of HIV. Priority actions, which support the targets and result areas, are generally aimed at national governments, though nearly all require support from non-government stakeholders. While priority actions may vary by geographic region, even in the context of limited resources, our consensus statement offers concrete recommendations regarding multimorbidity, HRQoL, and stigma and discrimination that all health systems should aspire to implement. The consensus points and next steps (Box 3) articulate the direction in which healthcare for PLHIV should evolve in all countries, emphasising that a reorientation of health systems will necessarily be incremental. The expert panel also recognises that the evidence base underpinning its conclusions is limited in some ways but rapidly growing. Thus the consensus statement should be regarded as an initial articulation of a transformational process, to be refined as stakeholders in government, civil society, research, healthcare and the social and legal sectors deepen their understanding of how health systems can support PLHIV in achieving optimal well-being.

Across the three Delphi rounds, three consensus points (2.4, 4.1, and 4.4) generated lower overall agreement than the others. This was reflected in our decision to continue to evaluate them on a four-point Likert scale in the third Delphi round instead of the binary agree/disagree measurement used with the other points. The results from that round revealed a continued, relatively lower level of agreement for two of the statements (2.4 and 4.1) (Table 3). The lack of unanimity here may highlight a lack of consensus among different types of stakeholders in the broader HIV field regarding whether some issues (e.g., pain) should be prioritized in health system efforts to improve health outcomes for PLHIV. It may also reflect the lack of shared conceptual frameworks for other issues such as stigma and discrimination, punitive laws, and intersectionality, pointing to a possible need to engage a wider range of stakeholders in dialog about these issues.

Box 3 Key next steps for health systems to advance the long-term well-being of people living with HIV 1. Incorporate the monitoring of comorbidities in electronic health records, where feasible, for use in integrated clinical care and international multimorbidity monitoring.

2. Develop and pilot models of care that employ frameworks for healthy aging, frailty, functional ability, and other dimensions of health that are relevant to PLHIV, using HRQoL as a key outcome measure. Meaningfully involve PLHIV in these efforts.

3. Expand integrated HIV and primary care outreach services to locations and times that reduce access barriers for marginalized and vulnerable groups. Pilot integrated models of care for these groups that link them with the formal health system, including community-based health and psychosocial services and peer support programs.

4. Establish annual surveys of PLHIV, conducted at the subnational and/or facility level, to collect and document data on HRQoL and on experiences of stigma and discrimination in healthcare settings.

5. Implement interventions to strengthen empathy among healthcare staff and decrease stigma and discrimination in healthcare settings. These should be accompanied by interventions involving peers and community members, which can reduce internalized stigma in PLHIV by enhancing the effect of protective factors such as empowerment, social support, resistance, and adaptive coping strategies.

### Limitations and strengths

The Delphi process involving a virtual expert panel enables the systematic gathering of the collective knowledge and recommendations of leaders in the field while separated by physical distances^27,121^. However, this process has both limitations and strengths. Typical of the Delphi method, the research team used purposive sampling to recruit potential expert panel members from existing HIV networks. The initial roster was then expanded using snowball sampling to include a wider range of experts and, potentially, viewpoints. The use of English in conducting the study may have restricted the composition of the expert panel and, by extension, the study outputs. The size of Delphi panels can range widely. This study included 44 individuals, which is within the 10 to 50 generally recommended for expert panels^122^. Members were from 22 countries across the six WHO regions. We note that 25% of them worked in a country different from their country of origin and speculate that their assessment of consensus points may have benefitted from their diverse backgrounds. However, this also meant that in the case of Africa, with its disproportionate burden of HIV, two of the six African members no longer worked on the continent. Thus, it is not clear to what extent they considered the African context in their assessment of consensus points. That the composition of the panel reflected activities spanning research, service provision, community-based care, policy, and advocacy provided the project with expertise and knowledge regarding the priorities of diverse stakeholder groups.

An advantage of using scoping reviews for the three domains is that expert panel members could draw on their knowledge and experience to focus on issues that pertain to improving the well-being of PLHIV but may not yet be extensively documented in the peer-reviewed or gray literature. A further strength of the study was the collection of open-ended (text-based) comments from respondents who agreed with statements (e.g., for further input and suggested edits), as well as from respondents who disagreed (e.g., to understand their rationale and suggested changes). This elicitation and incorporation of feedback from respondents across all three survey rounds (with high response rates, i.e., R1, R2 = 86%; R3 = 91%) and the online meeting were likely critical to the consistent increase in levels of agreement.

Finally, the worldwide relevance of the expert panel’s consensus is suggested by the fact that the consensus statement had been endorsed by 67 organizations (Box 2) at the time this article went to press.

## Conclusion

Prevention and treatment of HIV infection including access to ART remain a major public health and human rights challenge that requires urgent, sustained attention. This consensus statement addresses the concurrent role of health systems in advancing the long-term well-being of PLHIV. Multimorbidity, HRQoL, and stigma and discrimination continue to be major issues for PLHIV, including those who have achieved viral suppression and in particular those from marginalized populations.

The consensus statement was prepared by a large multidisciplinary panel of experts representing the interests of a diverse range of stakeholders including HIV community members. Its purpose was to facilitate consensus on major health-related issues affecting PLHIV to be addressed from a patient-centered perspective, noting that many issues are not captured in current HIV monitoring processes and guidelines. While additional research is needed, the burden of creating new, simple, standardized indicators for insufficiently addressed issues will largely fall on international bodies such as WHO and UNAIDS, both of which have mechanisms for community consultation and engagement. This process will require a strong commitment from all Member States, which must report on the indicators and implement policies to enhance health system performance. We encourage these organizations and national health system monitoring bodies to continue to take on this task, to report at regular intervals, and to meaningfully involve PLHIV, even when additional costs are involved. PLHIV and civil society organizations should also play a role in reporting ways in which governments are not meeting their obligations. Ultimately, a concerted effort by all stakeholders is required to ensure the long-term well-being of the millions of people around the world living with HIV and to end the HIV pandemic.

## Supplementary information

Supplementary Information
