# Supplementary material for: Consensus statement on the role of health systems in advancing the long-term well-being of people living with HIV
Source: Nat Commun. 2021 Jul 16;12:4450. doi: 10.1038/s41467-021-24673-w (PMC8285468; doi:10.1038/s41467-021-24673-w)
Supplement: Supplementary file 1 — Supplementary Information [file 41467_2021_24673_MOESM1_ESM.pdf]

Supplementary information

Supplementary File 1. Methodological details

<u>Consensus project coordination and expert panel members</u>

This consensus process concerning the long-term well-being of PLHIV followed from earlier publications and debate (Lazarus JV et al. *BMC Med,* 2016; *The Lancet HIV* review "Living well with HIV", 2019) regarding the need to move beyond a focus on viral suppression, previously deemed "the fourth 90," and to set the next key markers of successful HIV treatment that are not limited to the UNAIDS testing and treatment targets. The project secretariat (JL and KS) led the process that commenced with compiling a list of potential expert panel members comprised of HIV/AIDS clinicians, researchers, and health systems, advocacy and policy professionals and eliciting recommendations of additional participants from them (i.e., snowball sampling approach) for a total of 50 (see Tables 1 and 2); 44 individuals agreed to participate in the Delphi process. The expert panel was led by a Steering Committee (SC) comprised of 11 members (JA, RBL*, GB, GC, ND*, MJF, RH, AK*, JVL*, CS, KS) four of whom served as project co-chairs (denoted by *). Following preliminary consultations to set the parameters of the consensus project, the SC established working groups on the following three topics: multimorbidity (21 members; co-chairs: GB and AK); self-reported health-related quality of life (HRQoL) (7 members; co-chairs: MJF and RH); HIV-related stigma and discrimination (12 members; co-chairs: JA and GC). The remaining two expert panel members provided comprehensive review of the consensus document.

<u>Review of evidence</u>

Topic-specific scoping reviews were conducted by the respective working groups. This resulted in summaries from the working group co-chairs of the key elements from the reviews, which informed an initial draft of consensus points prepared by the secretariat. The points were revised following SC review in preparation for the first survey round of the Delphi process involving the full expert panel. Draft consensus points were organised under four domains in the survey including framing a comprehensive health agenda for PLHIV, multimorbidity, HRQoL, and HIV-related stigma, and discrimination.

<u>Delphi consensus development process</u>

The data collection stages consisted of a first (R1) and second (R2) survey round, and an online meeting of the expert panel, followed by a third and final (R3) survey. Data collection took place in November–December 2020. We used the QualtricsXM® platform to develop and distribute the survey rounds. The R1 survey contained 29 draft points with 4-point Likert-type categories for respondents to indicate their level of agreement/disagreement (i.e., Agree/Somewhat agree/Somewhat disagree/Disagree) with the points. For R1, responses of *agreement* led to an open-ended option for respondents to provide comments and/or suggest edits to the points, while responses of *disagreement* provided an additional option to include an explanation of or rationale for their disagreement. Based on expert panel suggestions to the points in R1, the revised R2 survey contained 33 points, reflecting recommendations that larger points be divided, as well as other substantive edits to the content. In the R2 survey, we included a brief summary of the edits made to each of the points so that respondents would have that information as they indicated their level of agreement or disagreement with them. The open-ended comment options were provided to all but those who responded 'Agree' with the points. The online meeting that occurred following the R2 survey provided an opportunity for all members of the expert panel to provide comments regarding the current consensus points as they had been revised to that point in the Delphi process. In addition, the secretariat led a discussion on select issues raised in the previous rounds that would most benefit from a real-time, interactive process. This led to preparation of the final set of 31

points for R3. The consensus points resulted in fairly high levels of agreement in R1 that generally increased in R2; thus, most points in R3 were presented with a binary (Agree/Disagree) response option. Only three points (2.4, 4.1, 4.4) on topics where there were substantially more comments remained with the four response categories. Finally, a text box was provided at the end of each of the four survey domain sections providing respondents with the option to include open-ended comments.

Scoring of consensus points

We present the level of agreement with the consensus points following a grading system recently used by others in a Delphi process focusing on obesity stigma (Rubino F, et al., 2020), which denoted unanimous (100%) agreement with a 'U,' 90% to 99% agreement with an 'A,' 78% to 89% agreement with a 'B,' and 67% (a supermajority) to 77% agreement with a 'C.' As summarised in Table 3, there was unanimous agreement with 22 points and greater than 90% agreement with the remaining 9 points.

Ethical Considerations

This study was exempted from ethical review by the Ethics Committee of the Hospital Clínic (Barcelona, Spain), which supervises the human research conducted at the Barcelona Institute for Global Health (ISGlobal).
